# The Bioavailability of Solid-State Fermented Feather Meal Using a Novel Feather-Degrading Bacterium *Bacillus velezensis* PN1 in Broilers

**DOI:** 10.3390/ani14223254

**Published:** 2024-11-13

**Authors:** Tung-Lung Kuo, Hen-Wei Wei

**Affiliations:** Department of Animal Science and Technology, National Taiwan University, Taipei 10672, Taiwan

**Keywords:** feather, solid-state fermentation, in vitro pepsin digestibility, bioavailability, broiler

## Abstract

In this study, an effective feather-degrading bacterium was isolated and the solid-state fermentation condition for feather degradation was optimized. The resulting fermented feather meal (FFM) was evaluated for its bioavailability in broilers. Four *Bacillus* strains were examined for feather degradation rates, with *Bacillus velezensis* PN1 exhibiting the highest rate (83.24%, *p* < 0.05). A 3 × 3 × 3 factorial design was used to test substrate moisture content (45%, 55%, 65%), temperature (27 °C, 37 °C, 47 °C), and incubation time (24, 48, 72 h) for optimized fermentation conditions. In vitro pepsin digestibility (IVPD) revealed a significant interaction between temperature and time (*p* < 0.05), and the optimal performance was achieved at 37 °C for 72 h, followed by 37 °C for 48 h. Considering production time and cost, FFM2 (produced with 65% moisture at 37 °C for 48 h) was further compared with FFM1 (produced under the same conditions but at a lower temperature of 27 °C), and commercial hydrolyzed feather meal (HFM). IVPD did not differ significantly between FFM1, FFM2, and HFM, as they all showed significantly higher digestibility compared to raw feathers (RFs). FFM1, as well as FFM2, had significantly higher histidine and lysine concentrations than RF (*p* < 0.05), while FFM2 had a significantly lower cysteine content (*p* < 0.05). Based on fermentation conditions and amino acid composition, FFM2 was considered more suitable for large-scale production and was used in a broiler growth trial. The inclusion of 5% FFM2 in the broiler diet did not significantly affect body weight at 35 days compared to the 5% HFM group (*p* > 0.05), although both groups showed significantly lower weight gain than the 5% fish meal (FM) group (*p* < 0.05). The feed conversion ratio and performance efficiency factor did not differ significantly between the FFM2, HFM, and FM groups (*p* > 0.05). In groups fed diets without supplemental crystalline amino acids, growth performance did not significantly differ between the HFM and FFM2 groups (*p* > 0.05), although both performed significantly worse than groups with amino acid supplementation. In conclusion, FFM produced by *B. velezensis* PN1 through solid-state fermentation enhances feather bioavailability in poultry and can completely replace HFM when included at 5% in broiler diets, offering a potential sustainable alternative for poultry nutrition on a commercial scale.

## Simple Summary

A feather-degrading bacterium, *Bacillus velezensis* PN1, was isolated, and solid-state fermentation conditions were optimized. The resulting fermented feather meal (FFM) was evaluated for bioavailability in broilers. *B. velezensis* PN1 showed the highest feather degradation rate, with optimal fermentation achieved at 37 °C for 48–72 h. FFM2, produced with 65% moisture at 37 °C for 48 h, was compared with FFM1 (produced at 27 °C) and commercial hydrolyzed feather meal (HFM). FFM2 was found to be more suitable for large-scale production due to its amino acid profile and fermentation efficiency. In a broiler growth trial, diets containing 5% FFM2 showed no significant difference in body weight, feed conversion ratio, or performance efficiency compared to the 5% HFM group (*p* > 0.05). However, both FFM2 and HFM groups showed lower weight gain than the 5% fish meal (FM) group (*p* < 0.05). Without supplemental amino acids, growth performance did not differ between FFM2 and HFM groups (*p* > 0.05). In conclusion, FFM produced by *B. velezensis* PN1 can completely replace HFM when included at 5% in broiler diets.

## 1. Introduction

In recent years, the poultry industry has experienced rapid growth due to the increasing global population, resulting in a steady rise in the production of feather waste [1,2]. Feathers comprise approximately 90% crude protein, mainly keratin, an insoluble and resilient protein. Keratin contains a high amount of cysteine, reinforced by disulfide bonds, hydrogen bonds, and hydrophobic regions, which makes it resistant to degradation by conventional proteases, rendering it indigestible by animals [3,4,5,6]. Traditionally, feathers are processed by a method involving high temperature and high pressure, which break the disulfide and hydrogen bonds to improve their digestibility [7]. Nonetheless, this process is energy-intensive and can lead to the degradation of certain amino acids, reducing their bioavailability [5,8]. Although hydrolyzed feather meal (HFM) is a common ingredient in poultry feed, its use is limited due to its imbalanced amino acid profile. Therefore, HFM needs additional supplementation with lysine, histidine, or other amino acids [9,10]. Biological methods, such as microbial fermentation for feather processing, are a low-cost and environmentally friendly alternative. Several studies have reported that protein hydrolysates produced through microbially fermented feathers or feather meal may possess potential bioactive properties, including microbial biomass protein, altered amino acid composition, and extracellular enzymes that enhance digestibility [11,12,13,14]. Most research on feather fermentation has focused on submerged fermentation (SMF), followed by vacuum or other drying methods, to concentrate the products [15]. Although these methods improve the nutritional value of feather proteins, the energy consumption during SMF and subsequent drying is comparable to that of high-temperature hydrolysis, making large-scale production impractical [14]. By contrast, solid-state fermentation (SSF) is a more economical and feasible approach. SSF can utilize waste materials or agro-industrial byproducts as substrates, making it a low-energy environmentally friendly process that significantly minimizes pollution and waste [15]. Despite this, few studies have focused on optimizing SSF conditions specifically for feather degradation and its application in animal feed, possibly due to challenges such as the difficulty of microbial survival in lower moisture environments or the need for extended fermentation times to achieve decomposition rates comparable to those in submerged fermentation. Many *Bacillus* strains exhibit strong proteolytic activity, suggesting their potential for screening strains capable of efficiently degrading feathers [7,11,12,13]. Therefore, this study aimed to isolate *Bacillus* strains capable of efficiently degrading feathers and optimize SSF conditions to produce fermented feather meal (FFM). Preliminary screening was conducted using in vitro digestibility assays, followed by in vivo metabolic trials in roosters for bioavailability evaluation. The potential of FFM as a substitute for fish meal (FM) or HFM in broiler diets was also explored.

## 2. Materials and Methods

### 2.1. Feather Collection

Feathers were collected from white broiler chickens at a slaughterhouse (Charming Food International Marketing Co., Ltd., Taichung City, Taiwan). The feathers were obtained after mechanical removal of water and drying at 55 °C for 48 h. Subsequently, feathers were stored frozen (−20 °C) until further experiments. In the SMF culture medium, the feathers were cut into 1 cm pieces. Whole feathers were used in SSF.

### 2.2. Screening of Feather-Degrading Bacterium

For bacterial enrichment, four different *Bacillus* spp. were cultured in tryptone soya broth medium (TSB, HIMEDIA^®^, Mumbai, India), and bacteria were enriched under culture conditions of 3% feathers (*w*/*v*) and 2.5% inoculation scale (*v*/*v*) with the culture medium containing 0.05% NaCl, 0.07% KH_2_PO_4_, 0.14% K_2_HPO_4_, and 0.01% MgSO_4_. The pH was adjusted to 7.0 and the cultures were incubated at 100 rpm and 37 °C for 48 h. At the end of fermentation, the medium with bacteria was centrifuged at 3000× *g*, and the supernatant was collected for subsequent measurements of colony count, pH, free amino acids, and total nitrogen content. For the colony count, the sample was diluted serially, plated on TSB with agar (TSA, HIMEDIA^®^, Mumbai, India), and cultured for 24 h. The pH value was measured by a pH meter (digital pH meter, HORIBA, Kyoto, Japan). The analysis of free amino acids followed the protocol of Moore and Stein [16]. The total nitrogen content of the supernatant was determined using the Kjeldahl method according to AOAC [17]. The process for feather degradability was according to Huang et al. [13], with modifications, utilizing suction filtration with filter paper (NO. 40 filter paper, Whatman^®^, Buckinghamshire, UK). The degradation rate of feathers was calculated using the following formula:

Degradation rate of feather (%) = (A − B − C)/A × 100(1)

where A is the feather’s weight before fermentation, B is the residue of fermented feathers, and C is the weight of the filter paper. The data were calculated based on the dry matter.

### 2.3. Identification of Bacterium

The selected strain was isolated and sub-cultivated consecutively in TSA at 37 °C for 24 h, and then sequenced by Genomics (New Taipei City, Taiwan). The DNA from purified colonies was extracted using the Total DNA extraction kit (Taco™ nucleic acid extraction reagents, GeneReach, Taichung City, Taiwan) with the assistance of the Taco Automatic Nucleic Acid Extractor. After DNA extraction, 16S rRNA was amplified by polymerase chain reaction (PCR) using the purified and separated bacterial genome and 16S rRNA primers. The PCR did not yield easily distinguishable results related to bacterial diversity; therefore, three different sets of universal primers (27F/1525R, 8F2/806R, and fD1modF/16S1RR-B) were employed for 16S rRNA amplification. The successfully amplified and longer PCR products were sequenced and subsequently analyzed. The nucleotide BLAST function of NCBI was utilized to compare the amplified bacterial sequence with those in the DNA database.

### 2.4. Multifactorial Design for Solid-State Fermentation Conditions

The experiment was conducted as a 3 × 3 × 3 multifactorial design, consisting of substrate moisture (45, 55 or 65%), temperature (27, 37 or 47 °C), and time (24, 48 or 72 h) with 2.5% inoculation scale (*v*/*w*). After fermentation, the protein digestibility in terms of in vitro pepsin digestibility (IVPD) was assessed. Considering the fermentation time and cost, a subsequent IVPD comparison was made between fermented feather meal at 27 °C (FFM1) or 37 °C (FFM2) with 65% substrate moisture for 48 h, HFM, and raw feather (RF).

The assay of IVPD was according to AOAC [17], using 0.2% pepsin/mL (porcine gastric mucosa; P6887, Sigma Aldrich, St. Louis, MO, USA). The contents were filtered after digestion using suction filtration with filter paper, and then the crude protein content was determined following the process of AOAC [17].

The protein digestibility of both methods was calculated using the following formula:

Protein digestibility (%) = (A − B)/A × 100(2)

where A is the crude protein content of the sample before digestion and B is the crude protein content of the residue.

### 2.5. Animal Management and Experimental Design

#### 2.5.1. Apparent Digestibility and Metabolizable Energy Assay

The procedures of the metabolic trial were modified based on Hong et al. [18,19]. Twenty-four 25-week-old Lohmann LSL-LITE roosters with an average weight of 1.8 kg were assigned to three dietary treatment groups and one feed-deprived group (eight birds per diet). The feed-deprived group was fed dextrose to estimate endogenous losses of amino acids, energy, and nitrogen levels. Roosters were randomly assigned to individual cages (0.30 × 0.45 m) and housed in an environmentally controlled room. After feather removal, a colostomy closed pouch (Lapack-Super C, Alcare, Tokyo, Japan) was placed around the cloaca to replace the surgical collection method to obtain contaminant-free excreta.

For the experimentation, the feed was withdrawn 48 h prior to feeding the test ingredients. Each bird was force-fed a dextrose solution (25 g/100 mL water) at 24 and 30 h after the feed was removed. For each diet, 25 g of FFM1, FFM2, and HFM were administered in 100 mL of water to all birds at 48 and 54 h after feed withdrawal. To estimate endogenous losses, the feed-deprived group was tube-fed dextrose solution at 48 and 54 h after the feed was withdrawn. The colostomy closed pouch of each bird was changed every 4 h until 96 h after feed withdrawal. The experimental procedures were approved by the Institutional Animal Care and Use Committee (IACUC) of the National Taiwan University (NTU-110-EL-00116).

All excreta samples were frozen immediately post collection and freeze-dried. Subsequently, an approximate analysis was carried out according to AOAC [17]. The amino acid composition was determined by the National Animal Industry Foundation (Technical Service Center, Pingtung City, Taiwan) [17,20].

The apparent digestibility was estimated using the following formula:

Apparent digestibility (%) = [(A × B − C × D)/(A × B)] × 100(3)

where A is nutrient concentration in feed (%), B is tube-feeding amount of feed (g), C is nutrient concentration in excreta (%), and D is total excrement (g).

The calculations of apparent metabolizable energy (AME), true metabolizable energy (TME), nitrogen-corrected apparent metabolizable energy (AMEn), and nitrogen-corrected true metabolizable energy (TMEn) were performed followed the description of Hong et al. [18].

#### 2.5.2. Broiler Growth Experiment

The experiment was conducted on three hundred 0-day-old Arbor Acres broiler chicks (average weight of 44 g), which were assigned to five dietary treatment groups. The treatment groups included three balanced dietary amino acid groups: 5% fish meal (FM), HFM, and FFM2. There were two additional imbalanced dietary amino acid groups consisting of 5% HFM and FFM2 with no addition of crystalline amino acids. Each group had six replicates (10 birds per pen) following the randomized complete block design and was housed in an environmentally controlled room. Except for the imbalanced dietary amino acid groups, birds were fed mash diets to meet the nutrient requirements following the Arbor Acres broiler chicken recommendations (Table 1, Table 2 and Table 3) during the starter (d 0–10), grower (d 11–24), and finisher (d 25–35) periods. On day 10, 24, and 35, after 4 h of feeding deprivation, the body weight (BW) and the feed residues within each replicate were recorded, and then the feed intake (FI), weight gain (WG), and feed conversion ratio (FCR) were calculated. For every period, the mortalities were recorded. Performance efficiency factor (PEF) was calculated using the following formula.

Performance efficiency factor = [(BW × Survival rate (%)) ÷ (FCR × Day of age)] × 100(4)

The experimental procedures were approved by the Institutional Animal Care and Use Committee (IACUC), the National Taiwan University (NTU-111-EL-00091).

### 2.6. Statistical Analysis

All data were subjected to a General Linear Model (GLM) analysis of variance (ANOVA) using SAS 9.4 (Statistical Analysis System, Version 9.4, for Windows 10, 2023, Cary, NC, USA). When the data showed significant differences (*p* < 0.05), Tukey’s honest significant difference (HSD) method was applied for pairwise comparisons of means among different treatment groups. In the multifactorial design of SSF, upon detecting an interaction effect (*p* < 0.05), subsequent comparisons determined the presence of significant differences among individual groups. In the absence of an interaction effect (*p* > 0.05), the existence of main effects for each factor was further investigated.

## 3. Results

### 3.1. Screening and Identification of Feather-Degrading Bacterium

**Figure 1:**
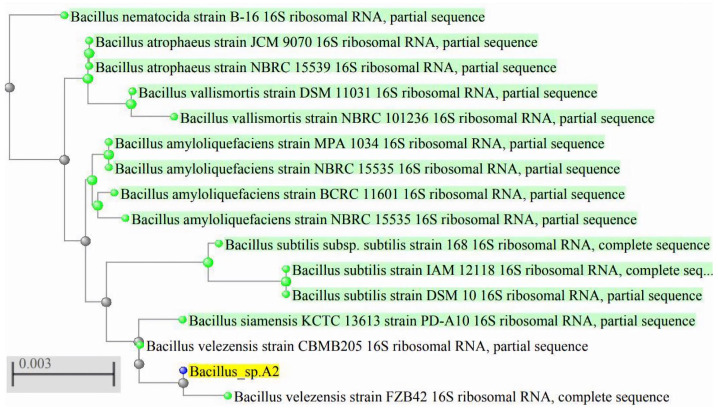
Phylogenetic tree based on 16S rDNA sequence comparisons of *Bacillus* sp. A2.

After 8 h of cultivation, all strains nearly reached a bacterial count of 8 log CFU/mL (Table 4). Strains A2 and A4 achieved bacterial counts exceeding 9 log CFU/mL after 48 h. The culture medium of all strains exhibited an increase in pH after fermentation, with the medium for strain A1 showing the highest pH (*p* < 0.05). The free amino acid content in the culture supernatant was highest for strain A2 at 0.65 mg/mL (*p* < 0.05). No significant differences were noted in the culture supernatant in terms of total nitrogen content among the strains (*p* > 0.05). Further, strain A2 exhibited the highest rate of feather degradation at 83.24% (*p* < 0.05). Based on the results in Table 4, strain A2 was identified as *Bacillus velezensis*, as shown in the phylogenetic tree in Figure 1, and was designated *B. velezensis* PN1 (PN1).

### 3.2. Multifactorial Design for Solid-State Fermentation Conditions

SSF conditions were optimized using in vitro protein digestibility (IVPD) as an indicator (Table 5). A significant interaction was observed between incubation temperature and time (*p* < 0.05). SSF at 37 °C for 72 h exhibited the best performance (*p* < 0.05), followed by 37 °C for 48 h. Considering the main effects, both substrate moisture content as well as incubation temperature had significant effects (*p* < 0.05), and the optimal conditions were 37 °C and 65% moisture content. For evaluating fermentation time and cost, subsequent experiments were performed to compare the IVPD of fermented feather meals (FFM1 and FFM2) prepared at either 27 °C or 37 °C with 65% moisture content for 48 h, commercial HFM, and raw feathers (RFs). The results (Table 6) indicated that the difference between the IVPD of HFM and FFM2 was not significant (*p* > 0.05), and the values for both were significantly higher than that of FFM1 (*p* < 0.05), with RF having the lowest IVPD (*p* < 0.05). The amino acid compositions of RF, FFM, and HFM are presented in Table 7. Considering the essential amino acids, HFM had the highest content (*p* < 0.05), while FFM showed a significant increase in histidine and lysine levels compared to those in RF (*p* < 0.05). Considering non-essential amino acids, cystine content was the lowest in HFM (*p* < 0.05), and FFM2 had a significantly lower content of cystine than that in RF (*p* < 0.05).

### 3.3. Apparent Digestibility and Metabolizable Energy Assay

The nutritional compositions of FFM and HFM are listed in Table 8. Compared to FFM, HFM had a higher moisture content, ether extract, ash, and gross energy (*p* < 0.05), while both types of FFM (FFM1 and FFM2) had a significantly higher crude protein content (*p* < 0.05). In terms of apparent digestibility, the digestibility of dry matter, organic matter, ether extract, and gross energy was higher for HFM compared to the two types of FFM (*p* < 0.05). In terms of metabolizable energy (Table 9), that of HFM was significantly higher than FFM (*p* < 0.05). Considering amino acid digestibility (Table 10), HFM exhibited generally better digestibility results, but the lysine digestibility of both types of FFM was significantly higher compared to that of HFM (*p* < 0.05).

### 3.4. Broiler Growth Experiment

The broiler diet was formulated based on the outcomes of previous experiments (Table 1, Table 2 and Table 3). The growth performance of broilers is presented in Table 11. The group fed a diet containing 5% FM exhibited the best BW and WG (*p* < 0.05), though there were no significant differences in FCR and PEF between the FM, HFM, and FFM2 groups (*p* > 0.05). Across all performance indicators, there were no significant differences between the HFM and FFM2 groups (*p* > 0.05). In groups not meeting the amino acid requirements, growth performance was significantly lower than in those with sufficient amino acid supplementation. However, the differences between the HFM and FFM2 groups lacking amino acid supplementation were not significant (*p* > 0.05).

## 4. Discussion

Feathers account for 5–7% of the body weight of poultry and comprise over 90% protein, with keratin making up approximately 90% of the total protein content. With the high cysteine content in the amino acid composition, feathers can serve as a poultry feed ingredient during feather growth. However, the disulfide bonds between keratin cysteine residues cannot be degraded by the digestive enzymes produced by poultry. Thus, to be used as a feed ingredient, feathers must first undergo hydrolysis. Current feather processing methods involve physical, chemical, and enzymatic hydrolysis or biological conversion. The limitations of the first three methods are protein denaturation, amino acid degradation, and the high cost of enzyme isolation and purification. In contrast, these drawbacks can be avoided through biological conversion methods [13,21].

For fermentation to occur, the microorganism must be capable of secreting keratinase, which must exhibit robust hydrolytic activity. Among various bacterial genera, most strains of *Bacillus* are well known for their strong extracellular protease secretion properties. However, the production of keratinase is influenced by the presence of keratin in the culture medium [22,23]. Therefore, in this study, feathers were used as the primary substrate in the SMF medium to assess the feather-degrading capabilities of four *Bacillus* strains to select the most appropriate strain (Table 4). After 48 h of fermentation, all strains achieved bacterial counts of 8 log CFU/mL, but those of strains A2 and A4 exceeded 9 log CFU/mL. In this context, the groups did not differ significantly, indicating that all four strains are capable of proliferating in a feather-based fermentation substrate. Feather degradation results in the production of free amino acids or NH_4_ lead to an increase in pH levels in the culture supernatant, a phenomenon also observed by Schwede et al. [24] and Peng et al. [25]. After fermentation, the total nitrogen content in the culture supernatant of strain A2 was higher than that of the other strains, although the difference was not significant (*p* < 0.1). Strain A2 exhibited a significantly higher feather degradation rate, reaching 83.24%, suggesting that, during SMF, this strain utilizes the feather substrate more effectively than the other three strains. Accordingly, strain A2 was selected for further experimentation. The 16S rRNA sequencing of strain A2 revealed the highest similarity to that of *B. velezensis* (Figure 1), and it was, therefore, subsequently designated *B. velezensis* PN1 (PN1).

Most studies on pure feather fermentation have focused on SMF, which involves a large amount of water, and the energy consumption for drying the fermentation liquid after large-scale production is comparable to that of traditional high-temperature and high-pressure methods, which limits its scalability [12,14,15]. In contrast, SSF offers more economical and practical advantages, although most of the literature focuses on keratinase production, and a few studies directly applied the fermented product to animal feed [15,26,27,28,29]. Considering these, the current study adopted the SSF method, and fermentation conditions such as optimal temperature, moisture, and duration of fermentation were determined beforehand, given that fermentation time directly affects production costs [30]. Additionally, the resulting product from feathers is positioned as a source of cystine for feather growth in poultry. Thus, indicators such as IVPD, changes in amino acid composition before and after hydrolysis, rooster digestibility of the final product, and its impact on the growth performance of broilers are key factors for evaluating the quality of HFM. Among these, while IVPD is the easiest to measure, it cannot ascertain whether excessive hydrolysis degraded the amino acids [8,9,31].

A factorial design was used in this study to explore the optimal fermentation conditions for FFM, with the variables being incubation temperature, substrate moisture, and fermentation time, and the preliminary screening indicator being IVPD. Temperature and moisture are critical for microbial growth in SSF [32]. As shown in Table 5, PN1 grew well at both 27 °C and 37 °C, but higher temperatures (47 °C) negatively impacted FFM digestibility. The optimal moisture content was 65%. Compared to SMF, microbial growth in low-free-water solid substrates yields less wastewater, thereby reducing the drying costs in downstream processes. The substrate also has lower water activity; hence, the energy required for sterilization is reduced and the risk of contamination is lowered [33]. However, while SSF has lower energy requirements and preserves amino acid integrity better than high-temperature hydrolysis, it incurs a higher time cost. The extended fermentation period limits throughput, which can reduce production capacity and raise operational costs in large-scale applications. These factors, combined with lower substrate loading per batch, present scalability challenges that must be addressed before SSF can fully replace traditional methods in high-capacity production settings. Table 5 reveals an interaction between temperature and time, with 37 °C for 72 h yielding the best results, although the outcome was not significantly different compared to 37 °C for 48 h. To balance fermentation time and cost, the treatment conditions of 27 °C or 37 °C with 65% moisture for 48 h were selected for subsequent trials, and the products were named FFM1 and FFM2, respectively. The IVPD was compared between the fermented products, RF, and HFM (Table 6), which showed that HFM had the highest IVPD, but there was no significant difference between HFM and FFM2, indicating the commercial potential of FFM2 in terms of digestibility. FFM1 as well as FFM2 had significantly higher IVPD than RF, with the difference between them being non-significant. Consistent with the results of this study, Bertsch and Coello [14] reported that feather fermentation with no high-temperature and high-pressure hydrolysis can improve pepsin digestibility in vitro to a level comparable to commercial feather meal. While feather meal obtained through high-temperature and high-pressure hydrolysis can improve in vitro digestibility, a risk of excessive hydrolysis exists that may reduce its nutritional value [8]. In this study, we employed SSF, which does not require extreme processing conditions leading to the degradation of amino acids. In microbial growth, the substrate can be decomposed during the synthesis of amino acids [34]. Therefore, we determined the amino acid composition of both the raw materials and the fermented products to assess the differences.

Compared to the amino acid composition of RF, significantly higher levels of all amino acids except tryptophan, cystine, proline, and serine were observed in HFM (Table 7). This discrepancy may be due to the source of HFM, which is derived not only from feathers but possibly also includes other poultry by-products such as blood, viscera, and fat trimmings [10,14,35,36,37]. Compared to RF, both FFM1 as well as FFM2 showed significantly lower levels of arginine, isoleucine, leucine, phenylalanine, valine, and alanine; yet, the concentrations of lysine and histidine increased significantly. This finding aligns with observations by Williams et al. [12], Bertsch and Coello [14], and Machado et al. [38], who indicated that the biomass produced by microorganisms during fermentation can also serve as a source of amino acids. This may also explain why HFM has a higher content of moisture, ether extract, ash, and total energy than FFM, while the crude protein content is higher in FFM (Table 8). The cystine content in FFM2 (7.72 g/16 N) was significantly lower than that of RF (8.56 g/16 N). Moritz and Latshaw [8] noted that the structural integrity of feathers is primarily maintained by the disulfide bonds of cystine in feather keratin. Therefore, appropriately lowering cystine content could enhance digestibility. With the addition of even 5% FFM2, the amino acid requirements for broiler diets can still be met. Consequently, for subsequent trials on broiler growth, FFM was prepared using the fermentation conditions established for FFM2. SSF provides an effective alternative to commercial hydrolysis by preserving amino acid integrity without the need for extreme processing conditions. This method minimizes protein and amino acid degradation, thereby reducing the risk of excessive hydrolysis that can diminish nutritional value. Unlike commercial hydrolysis, which can be cost-prohibitive due to high energy requirements, SSF leverages natural microbial activity to achieve comparable digestibility and bioavailability outcomes, making it a more economically viable and sustainable option for large-scale feed production.

Bryan and Classen [31] noted that, while in vitro digestibility can aid in the rapid assessment of the digestibility of ingredients, it does not correlate fully with the degree of digestion occurring within the gastrointestinal tract of an animal. Therefore, in this study, mature young roosters were used to evaluate the in vivo digestibility of the fermented products. Typically, this method involves surgical techniques [18,19,39,40]; however, in this study, a colostomy closed pouch was employed as a non-surgical alternative. HFM exhibited superior digestibility in terms of dry matter, organic matter, lipids, total energy, and most amino acids (Table 8 and Table 10). This enhanced digestibility may be due to the amino acid content, which influences the differences noted in digestibility and metabolizable energy (Table 8 and Table 9). Particularly, the digestibility of lysine was significantly higher in the two FFM treatments compared to HFM. This could be because HFM is not derived solely from feathers; moreover, the high-temperature and high-pressure hydrolysis process can result in varying levels of bioavailable amino acids depending on the specific conditions applied. Earlier, Moritz and Latshaw [8] opined that short-duration high-pressure hydrolysis can help preserve a greater proportion of bioavailable amino acids. While lysine content was higher in the HFM used in this study, its digestibility was lower, indicating that this is not solely a function of a single factor, but rather a cumulative outcome of the raw material source and the hydrolysis process employed. The results further indicated that the optimal fermentation temperature for PN1 in the SSF of feathers is likely between 27 and 37 °C (Table 5). As a result, FFM1 (cultured at 27 °C) and FFM2 (cultured at 37 °C) did not differ significantly in terms of IVPD, amino acid composition, or in vivo digestibility. However, during fermentation, the substrate can generate metabolic heat due to its decomposition, necessitating the design of the fermentation environment for the removal of this metabolic heat [32]. In large-scale SSF, lower temperatures for fermentation require the establishment of a cooling system to enhance metabolic heat removal rate, which can decrease substrate loading capacity and ultimately reduce economic efficiency [30]. Compared to the two FFM treatments, the lower cultivation temperature for FFM1 in large-scale fermentation may exacerbate these heat dissipation issues. Furthermore, during the SMF process, PN1 proliferated at 37 °C; therefore, reducing the cultivation temperature in large-scale SSF may lead to slower growth rates. Although no significant differences were noted between FFM1 and FFM2 across all parameters, these heat-related issues may be mitigated by FFM2. Additionally, a comparison of amino acid composition against RF reveals that FFM2 has advantageous qualities. Therefore, in subsequent growth trials with broiler chickens, FFM2 will be utilized.

The application of feather meal is inherently limited due to its low essential amino acid content and high non-essential amino acid content, restricting its incorporation into broiler diets to no more than 5% [41]. In this study, the growth performance of broilers fed diets containing 5% FM, HFM, and FFM2 were compared in addition to the distinction between the effects of HFM and FFM2 on broiler growth with no supplementation of the recommended levels of amino acids (Table 1, Table 2 and Table 3). FFM2, prepared from pure feathers, has a higher crude protein content (88.93%), allowing for a reduction in the inclusion of bulk protein sources, including soybean meal, while still necessitating the addition of crystalline amino acids to fulfill the nutritional requirements of broilers. Over the entire rearing period (Table 11), compared to FM, both FFM2 and HFM resulted in lower growth performance, particularly from days 0 to 10 and 11 to 24, and no significant differences were observed during days 25–35. Song et al. [42] observed that enzymatic hydrolysis and high-temperature and high-pressure processing, or expanding processes, do not enhance the digestibility of feathers in chicks, thus not promoting the use of feather meal in chick diets. Conversely, Safari et al. [21] reported that adding 4% solid-state FFM to broiler diets, along with the supplementation of amino acid requirements, led to a growth performance comparable to that of corn–soy meal diets during the 0–42 day period. For practical production strategies for fast-growing broilers, the inclusion of FM as a small protein supplement is suggested in the early and mid-rearing phases, while HFM, supplemented with crystalline amino acids, can be added later to meet nutritional needs. The study also demonstrates that, if crystalline amino acids are not provided, such as in both HFM and FFM2 groups, to meet amino acid requirements, deficiencies in certain essential amino acids may lead to significantly lower growth performance compared to groups receiving adequate amino acid supplementation. These findings indicate that, despite the limited incorporation of feather meal (only 5%), its use, whether through high-temperature and high-pressure processing or SSF, negatively impacts broiler growth due to its imbalanced amino acid composition. Therefore, the supplementation of deficient essential amino acids in feed formulations must be carefully considered [9,10]. However, there were no significant differences in growth performance between the two groups of chickens, indicating that, although the nutritional values of HFM, produced via high-temperature and high-pressure hydrolysis, are generally higher than those of FFM in terms of in vivo digestibility in roosters, the high digestibility of nutrients in HFM does not negate the negative impacts of amino acid imbalances when only 5% is included in broiler diets.

## 5. Conclusions

The strain selected for this study, *Bacillus velezensis* PN1, effectively decomposes poultry feathers. The optimal conditions for producing FFM through SSF are a moisture content of 65%, a cultivation temperature of 37 °C, and a fermentation time of 48 h. This FFM can be used in broiler diets at an inclusion rate of 5% during the fattening phase to supplement the diet with crystalline amino acids and meet the nutritional needs of broilers. This approach can completely replace HFM produced by high-temperature and high-pressure methods. The further optimization of SSF conditions and process parameters could improve feather utilization efficiency, making this method even more cost-effective and environmentally friendly, offering a potential sustainable alternative for poultry nutrition on a commercial scale. Future research could focus on refining the SSF process to enhance degradation rates, improve the amino acid profile, and increase the overall bioavailability of feather meal.

## Figures and Tables

**Table 1:** Nutrient composition (% as feed) of diets for broilers, 0–10 d ^1^.

Ingredients	Balance of Dietary Amino Acid			Imbalance of Dietary Amino Acid	
	FM	HFM	FFM2	HFM	FFM2
Corn meal	51.27	49.62	52.33	48.28	48.28
Soybean meal	37.15	37.67	34.33	35.59	35.59
Fish meal	5.00	-	-	-	-
HFM	-	5.00	-	5.00	-
FFM	-	-	5.00	-	5.00
Soybean oil	2.97	3.18	3.57	3.84	5.13
DL-Methionine	0.160	0.209	0.238	-	-
Lysine-HCl, 78%	0.115	0.254	0.396	-	-
Threonine	0.080	0.038	0.078	-	-
Glutamic acid	-	-	-	1.95	-
SiO_2_	-	-	-	1.30	1.95
Calcium carbonate	1.286	1.538	1.552	1.540	1.539
Ca(H_2_PO_4_)_2_	0.958	1.474	1.491	1.502	1.502
Salt	0.30	0.30	0.30	0.30	0.30
Mineral premix ^2^	0.20	0.20	0.20	0.20	0.20
Vitamin premix ^3^	0.30	0.30	0.30	0.30	0.30
Choline–chloride, 50%	0.15	0.15	0.15	0.15	0.15
Coccidiosis medicines	0.06	0.06	0.06	0.06	0.06
Total	100.00	100.00	100.00	100.00	100.00
Calculated value					
Crude protein	23.0	23.0	23.0	23.0	23.0
AMEn, kcal/kg	3000	3000	3000	3000	3000
Calcium	0.96	0.96	0.96	0.96	0.96
Available phosphorus	0.48	0.48	0.48	0.48	0.48
Methionine	0.56	0.56	0.56	0.34	0.32
Lysine	1.44	1.44	1.44	1.18	1.15
Threonine	0.97	0.97	0.97	0.89	0.90
Analyzed value (*n* = 3)					
Crude protein	23.3 ± 1.2	23.0 ± 0.8	23.2 ± 1.2	23.1 ± 1.1	23.2 ± 0.9

^1^ FM: fish meal; HFM: hydrolyzed feather meal; FFM2: solid-state fermented feather meal (fermented at 37 °C and 65% of moisture for 48 h). ^2^ Minerals supplemented per kg of diet: CuSO_4_·5H_2_O, 5.6 mg; ZnSO_4_·7H_2_O, 55.0 mg; MnSO_4_·H_2_O, 376.7 mg; Na_2_SeO_3_·5H_2_O, 0.7 mg; FeSO_4_·7H_2_O, 606.3 mg. ^3^ Vitamins supplemented per kg of diet: vitamin A, 11,000 IU; vitamin D, 5000 ICU; vitamin E, 75 IU, vitamin K3, 3.00 mg; thiamin, 3.00 mg; riboflavin, 8.00 g; niacin, 60.00 mg; vitamin B6, 4.00 mg; vitamin B12, 50.00 μg; folic acid, 2.00 mg; biotin, 0.17 mg; Ca-pantothenate, 15.00 mg.

**Table 2:** Nutrient composition (% as feed) of diets for broilers, 11–24 d ^1^.

Ingredients	Balance of Dietary Amino Acid			Imbalance of Dietary Amino Acid	
	FM	HFM	FFM2	HFM	FFM2
Corn meal	55.02	53.37	56.08	52.05	52.05
Soybean meal	33.09	33.61	30.27	31.40	31.40
Fish meal	5.00	-	-	-	-
HFM	-	5.00	-	5.00	-
FFM	-	-	5.00	-	5.00
Soybean oil	3.79	4.01	4.40	4.63	5.92
DL-Methionine	0.127	0.176	0.205	-	-
Lysine-HCl, 78%	0.049	0.188	0.329	-	-
Threonine	0.045	0.004	0.043	-	-
Glutamic acid	-	-	-	1.95	-
SiO_2_	-	-	-	1.29	1.95
Calcium carbonate	1.168	1.421	1.434	1.422	1.422
Ca(H_2_PO_4_)_2_	0.761	1.278	1.294	1.306	1.306
Salt	0.30	0.30	0.30	0.30	0.30
Mineral premix ^2^	0.20	0.20	0.20	0.20	0.20
Vitamin premix ^3^	0.30	0.30	0.30	0.30	0.30
Choline–chloride, 50%	0.15	0.15	0.15	0.15	0.15
Total	100.00	100.00	100.00	100.00	100.00
Calculated value					
Crude protein	21.5	21.5	21.5	21.5	21.5
AMEn, kcal/kg	3100	3100	3100	3100	3100
Calcium	0.87	0.87	0.87	0.87	0.87
Available phosphorus	0.43	0.43	0.43	0.43	0.43
Methionine	0.51	0.51	0.51	0.32	0.30
Lysine	1.29	1.29	1.29	1.08	1.05
Threonine	0.88	0.88	0.88	0.84	0.84
Analyzed value (*n* = 3)					
Crude protein	21.4 ± 1.2	21.4 ± 0.8	21.5 ± 0.3	21.4 ± 1.6	21.4 ± 1.7

^1, 2, 3^ As shown in Table 1.

**Table 3:** Nutrient composition (% as feed) of diets for broilers, 25–35 d ^1^.

Ingredients	Balance of Dietary Amino Acid			Imbalance of Dietary Amino Acid	
	FM	HFM	FFM2	HFM	FFM2
Corn meal	60.30	58.65	60.30	57.38	57.38
Soybean meal	27.53	28.01	27.53	25.77	25.77
Fish meal	5.00	-	5.00	-	-
HFM	-	5.00	-	5.00	-
FFM	-	-	-	-	5.00
Soybean oil	4.37	4.59	4.37	5.19	6.48
DL-Methionine	0.109	0.159	0.109	-	-
Lysine-HCl, 78%	0.054	0.197	0.054	-	-
Threonine	0.021	-	0.021	-	-
Glutamic acid	-	-	-	1.95	-
SiO_2_	-	-	-	1.29	1.95
Calcium carbonate	1.072	1.327	1.072	1.326	1.326
Ca(H_2_PO_4_)_2_	0.594	1.114	0.594	1.139	1.139
Salt	0.30	0.30	0.30	0.30	0.30
Mineral premix ^2^	0.20	0.20	0.20	0.20	0.20
Vitamin premix ^3^	0.30	0.30	0.30	0.30	0.30
Choline–chloride, 50%	0.15	0.15	0.15	0.15	0.15
Total	100.00	100.00	100.00	100.00	100.00
Calculated value					
Crude protein	19.5	19.5	19.5	19.5	19.5
AMEn, kcal/kg	3200	3200	3200	3200	3200
Calcium	0.79	0.79	0.79	0.79	0.79
Available phosphorus	0.39	0.39	0.39	0.39	0.39
Methionine	0.47	0.47	0.47	0.30	0.28
Lysine	1.16	1.16	1.16	0.96	0.92
Threonine	0.78	0.80	0.78	0.77	0.77
Analyzed value (*n* = 3)					
Crude protein	19.5 ± 0.4	19.7 ± 0.3	19.5 ± 1.6	19.4 ± 0.7	19.3 ± 0.5

^1, 2, 3^ As shown in Table 1.

**Table 4:** Effects of submerged fermented feather by different *Bacillus* spp. at 48 h ^1,2^.

Item	A1	A2	A3	A4	SEM	*p*-Value
Colony count, log CFU/mL	8.86	9.04	8.72	9.02	0.12	0.2679
pH value	8.04 ^a^	7.97 ^a,b^	7.89 ^b^	7.88 ^b^	0.03	0.0064
Free amino acid, mg/mL	0.60 ^b^	0.65 ^a^	0.61 ^b^	0.61 ^b^	0.01	0.0016
Total N of supernatant, mg/mL	2.79	3.51	3.02	3.22	0.17	0.0770
Degradation rate of feather, % DM	64.34 ^b^	83.24 ^a^	69.93 ^b^	69.23 ^b^	1.99	0.0009

^1^ *n* = 3. ^2^ CFU: Colony-forming unit. DM: Dry matter. ^a,b^ Means in the same row with the same superscripts are not different (*p* > 0.05).

**Table 5:** Effects of incubation temperature, moisture, and time in in vitro pepsin digestibility (%) of FFM by *Bacillus velezensis* PN1 ^1^.

Main Effect, *n* = 27																																
Temp (°C)											MO (%)												Time (h)									
27	37			47				SEM			45			55			65			SEM			24		48		72				SEM	
65.36 ^a,b^	67.89 ^a^			62.65 ^b^				1.02			62.82 ^b^			64.45 ^b^			68.63 ^a^			0.99			64.39		64.86		66.65				1.08	
Temperature × Moisture, *n* = 9											Temperature × Time, *n* = 9												Moisture × Time, *n* = 9									
MO	Temp									SEM	Time		Temp								SEM		MO	Time								SEM
	27		37			47							27			37		47						24		48			72			
45	63.89		63.53			61.05				1.57	24		65.96 ^b,c^			63.06 ^c^		64.14 ^b,c^			1.65		45	63.44		64.34			65.39			1.69
55	63.28		67.87			62.19					48		64.14 ^b,c^			68.71 ^a,b^		61.74 ^c^					55	62.45		62.80			69.34			
65	68.92		72.29			64.69					72		65.99 ^b,c^			71.91 ^a^		62.05 ^c^					65	62.57		66.21			71.16			
Temperature × Moisture × Time, *n* = 3																																
Mo/Time		Temp																												SEM		
		27										37										47										
		24			48				72			24			48				72			24		48			72					
45		65.54			64.23				61.91			60.32			65.45				64.80			64.46		57.68			61.01			2.41		
55		65.89			59.28				64.67			62.71			68.05				72.84			64.40		61.05			61.13					
65		66.46			68.92				71.39			66.15			72.61				78.10			63.57		66.48			64.01					
*p*-value		Temp					Mo				Time				Temp × Mo						Temp × Time			Mo × Time				Temp × Mo × Time				
		0.0001					<0.0001				0.1215				0.2600						0.0022			0.1122				0.6667				

^1^ FFM: Solid-state fermented feather meal. Temp: Temperature. Mo: Moisture. ^a–c^ Means with the same superscripts are not different (*p* > 0.05).

**Table 6:** The in vitro pepsin digestibility (%) of FFM, HFM, and RF ^1,2^.

Item	RF	FFM1	FFM2	HFM	SEM	*p*-Value
Pepsin digestibility (%)	22.59 ^c^	72.89 ^b^	74.65 ^a,b^	77.26 ^a^	0.66	<0.0001

^1^ *n* = 3. ^2^ RF = Raw feather. FFM1: Solid-state fermented feather meal (fermented at 27 °C and 65% of moisture for 48 h). FFM2: Solid-state fermented feather meal (fermented at 37 °C and 65% of moisture for 48 h). HFM: Hydrolyzed feather meal. ^a–c^ Means within a row with the same superscripts are not different (*p* > 0.05).

**Table 7:** Amino acid composition (g/16 g N) of RF, FFM, and HFM ^1,2,3^.

Item	RF	FFM1	FFM2	HFM	SEM	*p*-Value
Indispensable amino acid						
Arginine	7.35 ^b^	6.59 ^c^	6.47 ^c^	8.93 ^a^	0.11	<0.0001
Histidine	0.56 ^c^	0.74 ^b^	0.70 ^b^	1.02 ^a^	0.02	<0.0001
Isoleucine	5.43 ^b^	4.98 ^c^	4.88 ^c^	6.23 ^a^	0.08	<0.0001
Leucine	8.45 ^b^	7.88 ^c^	7.78 ^c^	9.84 ^a^	0.10	<0.0001
Lysine	2.11 ^c^	2.32 ^b^	2.28 ^b^	3.56 ^a^	0.03	<0.0001
Methionine	0.74 ^b^	0.76 ^b^	0.73 ^b^	1.30 ^a^	0.01	<0.0001
Phenylalanine	5.37 ^b^	4.68 ^d^	4.93 ^c^	6.04 ^a^	0.05	<0.0001
Threonine	4.22 ^b^	4.25 ^b^	4.19 ^b^	4.84 ^a^	0.07	0.0004
Tryptophan	0.42 ^ab^	0.40 ^ab^	0.38 ^b^	0.54 ^a^	0.03	0.0331
Valine	7.87 ^b^	7.12 ^c^	7.21 ^c^	8.52 ^a^	0.10	<0.0001
Dispensable amino acid						
Alanine	4.82 ^b^	4.18 ^c^	4.18 ^c^	6.04 ^a^	0.06	<0.0001
Aspartic acid	6.91 ^b^	6.70 ^b^	6.62 ^b^	8.66 ^a^	0.07	<0.0001
Cystine	8.56 ^a^	8.12 ^ab^	7.72 ^b^	3.27 ^c^	0.18	<0.0001
Glycine	7.81 ^b^	7.53 ^b^	7.59 ^b^	10.83 ^a^	0.17	<0.0001
Glutamic acid	10.77 ^b^	10.85 ^b^	11.02 ^b^	13.97 ^a^	0.16	<0.0001
Proline	6.73	6.84	6.88	7.85	0.33	0.1326
Serine	8.92 ^ab^	8.46 ^b^	8.11 ^b^	9.48 ^a^	0.19	0.0049
Tyrosine	2.19 ^b^	2.31 ^b^	2.36 ^b^	2.92 ^a^	0.11	0.0055

^1^ *n* = 3. ^2^ RF, FFM1, FFM2, and HFM are as shown in Table 6. ^3^ The data were calculated based on dry matter. ^a–d^ Means within a row with the same superscripts are not different (*p* > 0.05).

**Table 8:** The nutrient composition and the apparent digestibility of FFM and HFM ^1,2^.

Item	FFM1	FFM2	HFM	SEM	*p*-Value
Approximate analysis, *n* = 3					
Moisture, %	2.81 ^b^	3.28 ^b^	11.85 ^a^	0.87	0.0005
Dry matter, %	97.19 ^a^	96.72 ^a^	88.15 ^b^	0.87	0.0005
Crude protein, %	89.89 ^a^	88.93 ^a^	71.28 ^b^	1.61	0.0003
Ether extract, %	5.21 ^b^	4.40 ^b^	16.58 ^a^	0.20	<0.0001
Crude fiber, %	0.27 ^b^	0.28 ^b^	0.96 ^a^	0.08	0.0011
Ash, %	1.06 ^b^	1.22 ^b^	2.16 ^a^	0.19	0.0115
Gross energy, kcal/kg	5802.12 ^b^	5639.11 ^c^	6352.17 ^a^	20.37	<0.0001
Apparent digestibility of ingredients, %, *n* = 8					
Dry matter	22.39 ^b^	24.97 ^b^	41.57 ^a^	0.88	<0.0001
Organic matter	23.05 ^b^	25.84 ^b^	35.73 ^a^	0.97	<0.0001
Ether extract	29.50 ^b^	33.31 ^b^	77.23 ^a^	2.84	<0.0001
Crude fiber	52.73	53.23	55.80	8.44	0.9627
Gross energy	34.09 ^b^	35.06 ^b^	56.68 ^a^	1.67	<0.0001

^1^ FFM1, FFM2, and HFM are as shown in Table 6. ^2^ The data were calculated based on dry matter. ^a–c^ Means within a row with the same superscripts are not different (*p* > 0.05).

**Table 9:** The metabolizable energy content (kcal/kg) of FFM and HFM ^1,2,3,4^.

Item	FFM1	FFM2	HFM	SEM	*p*-Value
AME	2066.14 ^b^	2088.27 ^b^	3600.53 ^a^	102.88	<0.0001
TME	2139.04 ^b^	2163.31 ^b^	3680.55 ^a^	102.88	<0.0001
AMEn	2066.00 ^b^	2088.14 ^b^	3600.44 ^a^	102.88	<0.0001
TMEn	2135.29 ^b^	2159.47 ^b^	3676.50 ^a^	102.88	<0.0001

^1^ *n* = 8. ^2^ FFM1, FFM2, and HFM are as shown in Table 6. ^3^ AME: Apparent metabolizable energy. TME: True metabolizable energy. AMEn: Nitrogen-corrected apparent metabolizable energy. TMEn: Nitrogen-corrected true metabolizable energy. ^4^ The data were calculated based on dry matter. ^a,b^ Means within a row with the same superscripts are not different (*p* > 0.05).

**Table 10:** The apparent amino acid digestibility (%) of FFM and HFM ^1,2,3^.

Item	FFM1	FFM2	HFM	SEM	*p*-Value
Indispensable amino acid					
Arginine	42.89 ^b^	43.23 ^b^	80.78 ^a^	0.75	<0.0001
Histidine	58.69	55.65	59.99	2.51	0.4678
Isoleucine	40.72 ^c^	45.30 ^b^	79.92 ^a^	1.04	<0.0001
Leucine	45.33 ^b^	45.47 ^b^	76.99 ^a^	0.75	<0.0001
Lysine	64.96 ^a^	65.54 ^a^	49.60 ^b^	1.04	<0.0001
Methionine	73.48	69.64	71.50	1.41	0.1829
Phenylalanine	40.55 ^b^	44.09 ^b^	75.36 ^a^	1.80	<0.0001
Threonine	38.96 ^b^	36.43 ^b^	66.45 ^a^	0.99	<0.0001
Tryptophan	93.05 ^a^	82.88 ^b^	98.05 ^a^	2.83	0.0036
Valine	35.16 ^c^	41.08 ^b^	78.06 ^a^	0.93	<0.0001
Dispensable amino acid					
Alanine	39.97 ^b^	40.84 ^b^	73.69 ^a^	0.92	<0.0001
Aspartic acid	39.92	40.76	43.57	1.53	0.2339
Cystine	37.82 ^a^	29.99 ^b^	44.59 ^a^	1.90	<0.0001
Glutamic acid	45.25 ^b^	45.40 ^b^	65.92 ^a^	0.92	<0.0001
Proline	29.84 ^b^	35.80 ^b^	62.99 ^a^	2.18	<0.0001
Serine	32.57 ^b^	33.69 ^b^	69.60 ^a^	1.25	<0.0001
Tyrosine	52.83 ^b^	53.94 ^b^	73.98 ^a^	1.44	<0.0001

^1^ *n* = 8. ^2^ FFM1, FFM2, and HFM are as shown in Table 7. ^3^ The data were calculated based on dry matter. ^a–c^ Means within a row with the same superscripts are not different (*p* > 0.05).

**Table 11:** The effects of solid-state fermented feather meal by *Bacillus velezensis* PN1 on the growth performance of broilers ^1^.

Period, Day	Balance of Dietary Amino Acid						Imbalance of Dietary Amino Acid				SEM	*p*-Value
	FM	*n*	HFM	*n*	FFM2	*n*	HFM	*n*	FFM2	*n*		
Body weight, BW, g/bird												
0	44	60	44	60	44	60	44	60	44	60	0.09	0.9998
10	281 ^a^	60	259 ^b^	60	255 ^b^	60	180 ^c^	60	191 ^c^	60	3.15	<0.0001
24	1138 ^a^	59	1060 ^b^	60	1059 ^b^	59	678 ^c^	60	701 ^c^	59	11.85	<0.0001
35	2128 ^a^	58	2002 ^b^	59	2028 ^b^	59	1271 ^c^	60	1267 ^c^	59	21.42	<0.0001
Feed intake, FI, g/bird												
0–10	268 ^a^	6	255 ^a^	6	250 ^a^	6	182 ^b^	6	202 ^b^	6	7.29	<0.0001
11–24	1144 ^a^	6	1144 ^a^	6	1152 ^a^	6	798 ^b^	6	801 ^b^	6	23.16	<0.0001
25–35	1552 ^a^	6	1511 ^a^	6	1540 ^a^	6	1121 ^b^	6	1111 ^b^	6	32.58	<0.0001
0–35	2964 ^a^	6	2910 ^a^	6	2943 ^a^	6	2102 ^b^	6	2114 ^b^	6	51.52	<0.0001
Weight gain, WG, g/bird												
0–10	237 ^a^	60	215 ^b^	60	211 ^b^	60	135 ^c^	60	146 ^c^	60	3.14	<0.0001
11–24	856 ^a^	59	801 ^b^	60	804 ^b^	60	499 ^c^	60	509 ^c^	60	10.37	<0.0001
25–35	991 ^a^	58	941 ^a^	59	969 ^a^	59	593 ^b^	60	566 ^b^	59	13.58	<0.0001
0–35	2084 ^a^	58	1957 ^b^	59	1984 ^b^	59	1227 ^c^	60	1222 ^c^	59	21.40	<0.0001
Feed conversion ratio, FCR, FI/WG												
0–10	1.13 ^b^	6	1.19 ^b^	6	1.19 ^b^	6	1.35 ^a^	6	1.38 ^a^	6	0.04	0.0002
11–24	1.33 ^b^	6	1.43 ^b^	6	1.43 ^b^	6	1.60 ^a^	6	1.57 ^a^	6	0.03	<0.0001
25–35	1.57 ^b^	6	1.61 ^b^	6	1.59 ^b^	6	1.89 ^a^	6	1.97 ^a^	6	0.03	<0.0001
0–35	1.42 ^b^	6	1.49 ^b^	6	1.48 ^b^	6	1.71 ^a^	6	1.73 ^a^	6	0.02	<0.0001
Survival rate, %												
0–35	96.67	6	98.33	6	98.33	6	100.00	6	98.33	6	1.65	0.7289
Performance efficiency factor, PEF												
0–35	413 ^a^	6	379 ^a^	6	385 ^a^	6	212 ^b^	6	206 ^b^	6	9.46	<0.0001

^1^: FM = Fish meal. HFM = Hydrolyzed feather meal. FFM2 = Fermented feather meal. ^a–c^: Means in the same row with different superscripts are significantly different (*p* < 0.05).

## Data Availability

The datasets used and/or analyzed during the current study are available from the corresponding author on reasonable request.
